# Spatial analysis of vaccine coverage in children under the age of 1 year by mesoregions in Paraíba a northeastern Brazilian state

**DOI:** 10.1371/journal.pone.0288651

**Published:** 2023-07-18

**Authors:** Nairmara Soares Pimentel Cunha, Ricardo Alves de Olinda, Sylvia Costa Lima Fahrat, Carolina Luisa Alves Barbieri, Alfésio Luís Ferreira Braga, Ysabely de Aguiar Pontes Pamplona, Lourdes Conceição Martins

**Affiliations:** 1 Vaccine Observatory, Stricto Sensu Graduate Program in Collective Health of the Catholic University of Santos, Santos, São Paulo, Brazil; 2 Children’s Institute, University Clinics Hospital, Faculty of Medicine, University of São Paulo, São Paulo, Brazil; 3 Department of Statistics, State University of Paraíba (UEPB), Paraíba, Brazil; 4 Environmental Exposure and Risk Assessment Group, Stricto Sensu Graduate Program in Collective Health of the Catholic University of Santos, Santos, São Paulo, Brazil; UFSJ: Universidade Federal de Sao Joao del-Rei, BRAZIL

## Abstract

Immunization is one of the most effective measures in public health, and it is responsible for the reduction of vaccine-preventable diseases. In the present study, vaccine coverage (VC) and the spatial dynamics of homogeneity of VC (HVC) were compared and analyzed in the terms of the immunobiologicals administered to children aged < 1 year in a state in Paraíba, Brazil. This is a mixed ecological study that used public-domain secondary data from the years 2016 and 2017 from the Information System of the Brazilian National Immunization Program (SI-PNI) and the Brazilian National Information System of Live Births (SINASC). VC rates were calculated by dividing the number of administered doses by the number of live births. Then, VC was classified into four categories. The Municipal HVC was considered adequate when the overall VC exceeds 75%. The study included a descriptive analysis and a spatial autocorrelation analysis for HVC using global and local Moran’s statistics. The stratified VC analysis revealed a significant number of municipalities in each of the state’s mesoregions with low or very low VC rates for all immunobiologicals, with the Mata Paraibana mesoregion having the worst percentages in both years studied. The spatial analysis of HVC revealed several clusters of inadequate homogeneity, with Mata Paraibana being the worst mesoregion in 2016. The analysis of spatial dynamics and spatial statistics techniques allows the precise identification of vulnerable areas, “vaccination pockets,” making it possible to develop plans aimed at meeting the targets of the PNI.

## Introduction

Immunization is the most efficient method to improve health indicators and reduce diseases preventable by vaccination during childhood. However, it is essential to ensure universal access to immunization for these benefits to occur. Monitoring inequality is an efficient way to ensure vaccination equality because all people should have access to the benefits of vaccination regardless of geographic location, ethnicity, socioeconomic level, age, gender, schooling, or special needs. The World Health Organization created a handbook on how to monitor vaccination inequality in order to help different countries meet the targets outlined in the document *Immunization Agenda 2030*: *A Global Strategy to Leave No One Behind* [1], stipulating a 90% target immunization rate at the national level and 80% at district or administrative sub-unit levels [2].

In Brazil, the National Immunization Program provides all vaccines deemed cost-effective for public health to the Brazilian population [3]. These vaccines are provided in the basic healthcare units of the Brazilian Unified Health System (SUS), thereby opting for a form of preventive care to promote and protect health [4].

The evaluation of vaccine coverage (VC) considers specific targets that must be met for each immunobiological agent, ranging from 90% for Bacillus Calmette–Guérin (BCG) and rotavirus to 95% for diphtheria–pertussis–tetanus (DPT), *Haemophilus influenzae* type B (HiB), poliomyelitis, pneumococcal, and meningococcal vaccines [5]. In contrast, the evaluation of the homogeneity of VC (HVC) within each municipality considers two SUS management instruments, the Organizing Contract of Public Actions in Health (COAP), whose results are considered adequate for a municipality when the established target is met for 75% or more of the vaccines analyzed [6], and the Qualification Program for Actions in Health Surveillance (PQA-VS), which considers HVC adequate when 100% of the vaccines analyzed in a given municipality meet the established target [7, 8].

Additionally, the Information System of the Brazilian National Immunization Program (SI-PNI) considers intermunicipality HVC adequate when at least 70% of the evaluated municipalities meet the established target [6, 7, 9].

According to Cutts *et al*. [10] and Sato [11], knowing which regions failed to meet the PNI targets is indispensable for PNI’s success in public health. Moreover, it is fundamental to measure VC and identify vulnerable areas for immunization programs to plan actions and thus prevent possible epidemics of vaccine-preventable diseases [12].

Paraíba is a state located in Brazil’s northeastern region, with a population of 4,059,905 in 2020 distributed in an area of 56,467.242 km^2^ [13]. The state as a whole has a high VC, but intermunicipality HVC does not meet the target of 70% of all municipalities in Paraíba having adequate coverage for each immunobiological agent [14].

**Fig 1:**
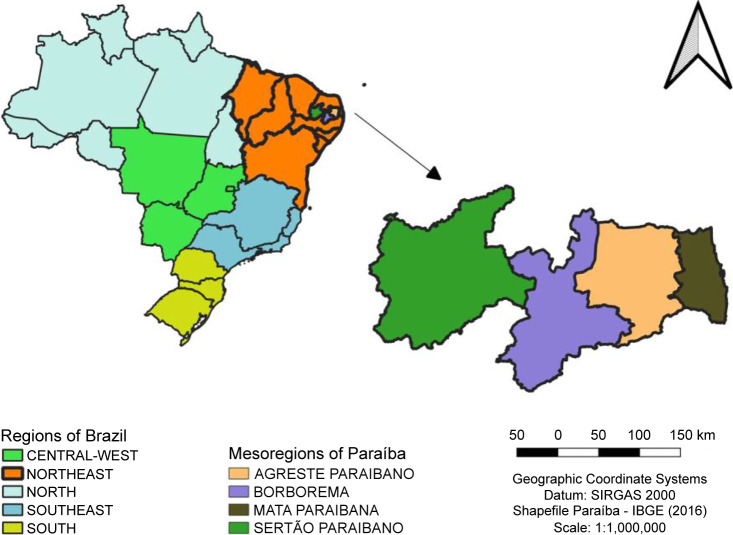
Map of the Brazil and mesoregions of Paraíba.

The regionalization of healthcare in Paraíba is defined in Resolution CIB 13/2015, and this resolution was used in this article. It categorizes the state’s 223 municipalities into four mesoregions: Mata Paraibana, Agreste Paraibano, Borborema, and Sertão Paraibano (Fig 1) [15].

Mata Paraibana comprises 30 municipalities and occupies 9.3% of the state area, but it is the mesoregion with the greatest population density and urbanization rate. Agreste Paraibano has 66 municipalities, Borborema has 44, and Sertão Paraibano has 83 municipalities. These mesoregions comprise 22.9%, 27.6%, and 40.3% of the state territory, respectively. Mata Paraibana is the mesoregion with the largest population (37.7%), followed by Agreste Paraibano with 31.8%, Sertão Paraibano with 22.6%, and Borborema with 7.9% [16].

Each mesoregion has its own characteristics: Mata Paraibana is characterized by sugarcane cultivation and is home to the majority of the state’s industries. Agreste Paraibano is predominantly agricultural (cotton, corn, cattle, sisal, and sugarcane). Borborema combines sisal, cotton, and cattle farming with mining, while livestock activities and temporary cultures predominate in Sertão Paraibano [17].

Spatial epidemiology is useful in identifying geographic areas and population groups at risk of disease or early death, and it also shows that the risk factors influencing the population’s health are not the same for all population groups [18]. Therefore, spatial analysis is an important tool for identifying spatial and spatial–temporal clusters in order to determine which areas are more vulnerable to health hazards [19].

Thus, the objective of the present study include the comparison of VC between the mesoregions and a spatial analysis of HVC by mesoregions of the State of Paraíba in children aged < 1 year in the period between 2016 and 2017.

## Materials and methods

This is a mixed ecological study, with the municipality of residence serving as the analysis unit [20] that used secondary data from the public domain from 2016 to 2017. We declare that the data used are from the public domain health and were obtained according to the criteria of good research practice and ethical precepts. The datasets analyzed during the current study are available in the Zenodo Data repository [https://doi.org/10.5281/zenodo.7478358].

The data were gathered and analyzed without identifying the subjects to maintain the privacy and confidentiality of the information, in accordance with the guidelines of the National Health Council n° 466/2012 [21], 510/2016 [22], and 580/2018 [23] with regard to research involving human participants that emphasizes dignity and respect for the research subjects.

VC was calculated by dividing the number of vaccine doses administered by the number of live births. The number of doses applied was obtained from the SI-PNI. The number of live births was obtained from the Brazilian National Information System of Live Births (SINASC), which is maintained by the SUS Department of Information Technology (DATASUS) and has a publicly accessible database.

The analysis unit used was the municipality, and the following immunobiological agents were analyzed: BCG, hepatitis B vaccine (HepB), meningococcal C vaccine (MnCc), DPT, HiB, poliomyelitis vaccine (Polio), rotavirus vaccine (Rota), and pneumococcal vaccine (Pneumo).

After VC was calculated, it was classified into four categories: very low (less than 50%), low (greater than or equal to 50% but less than the target), adequate (greater than or equal to the target but less than 120%), and high (greater than or equal to 120%). For BCG and rotavirus vaccines, the recommended target is 90%, while for DPT, HepB, poliomyelitis, pneumococcal, and meningococcal vaccines, the target is 95% [7].

HVC within a given municipality was calculated by dividing the number of vaccines with adequate VC in that municipality (according to the parameters defined for each vaccine by the PNI) by the total number of vaccines in that municipality and then multiplying the quotient by 100. Homogeneity proportions of <75% were considered inadequate.

Thematic maps were made to categorize VC as described above. These maps were made via geoprocessing using a geographic information system, spatial analysis techniques, and thematic cartography. The cartographic base containing the digital geodata of the municipalities was obtained from the Brazilian Institute of Geography and Statistics (IBGE) [24] using geographic projection and IBGE’s SIRGAS 2000 geodesic reference system. The software used was QGIS, version 2.18.

The Moran’s Index was calculated to analyze the spatial HVC patterns in children aged less than 1 year within a municipality. Spatial correlations were demonstrated using the Local Index of Spatial Association (LISA), also known as the local Moran’s Index. Clusters were identified using LISA maps and Moran’s maps. These analyses were conducted using GeoDa software, version 1.8.

Moran’s maps allow spatial dependence to be viewed. In these maps, the high/high and low/low classifications show positive spatial dependence. In the case of the present study, high/high refers to municipalities with high HVC, while low/low refers to municipalities with low HVC, with both cases being surrounded by neighboring municipalities with similar values. In contrast, high/low and low/high are spots of negative spatial association because the neighbors show divergent values. One of the tools most widely used in spatial analysis to assess the spatial correlation between areas is the global Moran’s Index. However, to produce local indicators to examine spatial patterns on a more detailed scale and thereby identify “pockets” of spatial dependence that cannot be viewed using the global index [25], LISA is calculated, making it possible to identify regions with significant spatial correlation [26].

In this study, the first-order “W” neighborhood matrix was used, where cities sharing a common physical boundary were considered neighbors. Adjacent regions are thought to be closely related than more distant ones, and these connections are represented using matrix cells, where the values 1 and 0 indicate the presence and absence of a common border. A 5% significance level was adopted.

Descriptive and inferential analyses were performed using SPSS software, version 24.0 (IBM Corporation, Armonk, NY, USA). A 5% significance level was adopted.

## Results

**Fig 2:**
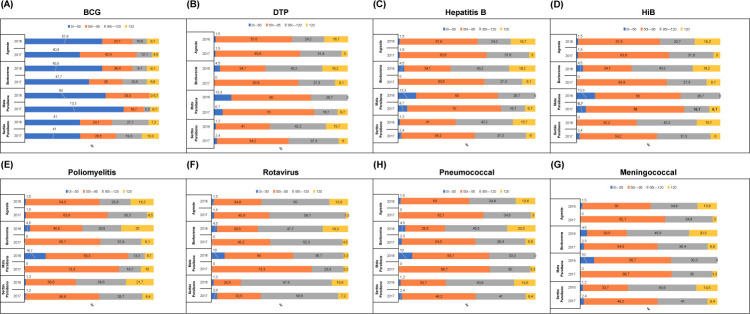
Vaccination coverage for children under a year old by Paraíba mesoregions, Brazil, 2016–2017. (A) Bacillus Calmette–Guérin, (B) diphtheria–pertussis–tetanus, (C) Hepatitis B, (D) *Haemophilus influenzae* type B, (E) Poliomyelitis, (F) Rotavirus, (G) Pneumococcal, (H) Meningococcal.

The plots show the percentage distribution of VC in 2016 and 2017 in the four mesoregions of Paraíba. Municipalities with very low VC are shown in blue, low VC in orange, adequate VC in gray, and high VC in yellow (Fig 2).

In Fig 2A, it can be observed that all mesoregions of Paraíba failed to meet an adequate VC for BCG, falling extremely short of the set target of 90%. In 2016, no municipality in the Mata Paraibana mesoregion presented adequate coverage, and in 2017, it had a very small percentage (3.3%). This is the mesoregion in which the state capital city of João Pessoa is located.

From 2016 to 2017, there was a decrease in the percentage of municipalities with high VC in all four mesoregions. However, this decrease was not observed in the adequate VC range, except in the Borborema mesoregion.

Fig 2B–2D show the DPT, HepB, and HiB immunobiological agents together because they are administered simultaneously in the form of a pentavalent vaccine. The plots demonstrate that there was a percentage drop in adequate VC in the Borborema, Mata Paraibana, and Sertão Paraibano mesoregions as well as an increase in the low VC category in the years analyzed. The high VC category shows a percentage drop, but this is not observed in the adequate VC category, except in the Mata Paraibana mesoregion, where adequate VC increased in percentage.

The poliomyelitis VC (Fig 2E) is concerning because less than 50% of the Paraíba municipalities had adequate coverage for this vaccine in both years studied. In the Mata Paraibana mesoregion, 63.3% of the municipalities were in the low VC category in 2016 and 73.3% in 2017. These are significantly high percentages. It can also be observed that there was a percentage increase between the years studied in the municipalities with adequate VC, except in the Sertão Paraibano mesoregion, where there was a percentage drop.

In terms of rotavirus VC (Fig 2F), only the Sertão Paraibano mesoregion had adequate VC in more than 50% of its municipalities. The Mata Paraibana mesoregion showed a 13.4% decrease in the adequate VC category in the years studied, and Sertão Paraibano had a 10.9% decrease. The percentage of municipalities with high VC decreased significantly in the years studied, except in Mata Paraibana, and all mesoregions showed an increase in the number of municipalities with low VC.

In terms of pneumococcal VC (Fig 2G), less than 50% of the municipalities in all mesoregions met the PNI goals for adequate VC, except in Sertão Paraibano in 2016, where 56.6% of this mesoregion’s municipalities had adequate VC. The Agreste Paraibano and Sertão Paraibano mesoregions had no municipalities with very low VC in 2016, but these municipalities demonstrated increases of 1.5% and 2.4%, respectively, in 2017. In contrast, the Borborema and Mata Paraibana mesoregions showed a decrease in the two years studied, reaching zero municipalities with very low VC in 2017.

**Fig 3:**
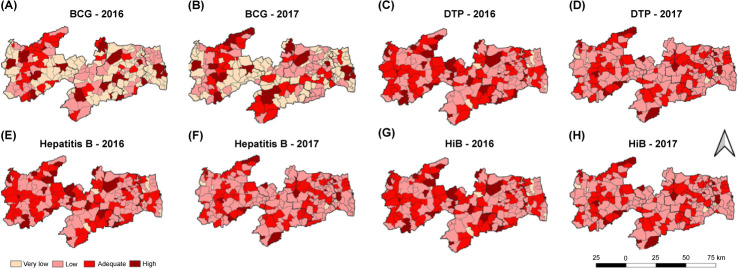
Vaccine coverage for BCG, DTP, HepB, and HiB by mesoregion of Paraíba, Brazil, 2016–2017. Bacillus Calmette–Guérin, (A, B) diphtheria–pertussis–tetanus, (C, D) Hepatitis B, (E, F) *Haemophilus influenzae* type B.

**Fig 4:**
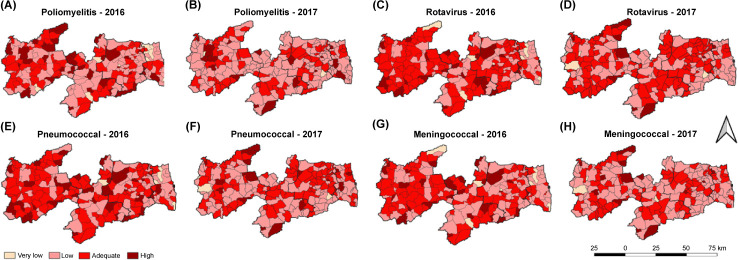
Vaccination coverage for polio, rotavirus, pneumococcal, and meningococcal vaccines by Paraíba mesoregions, Brazil, 2016–2017. Poliomyelitis, (A, B) Rotavirus, (C, D) Pneumococcal, (E, F) Meningococcal (G, H).

Fig 2H shows that all mesoregions failed to meet the PNI target for adequate meningococcal VC. The number of municipalities in the low VC category increased for MnCc in the years studied in all mesoregions. Figs 3 and 4 show the spatial dynamics of the immunobiologicals studied.

In Fig 3A and 3B [BCG], a cluster of municipalities with adequate VC can be observed in the Sertão Paraibano mesoregion in both studied years, as well as a cluster in the Southern Borborema mesoregion in 2017. Additionally, several clusters of municipalities with low and very low VCs can be seen in all mesoregions in both years. The state capital city, João Pessoa, located in the Mata Paraibana mesoregion, presents a high VC but is surrounded by areas with low or very low VC and another single municipality with a high VC.

In terms of DPT/HepB/HiB VC (Fig 3C–3H), some clusters of municipalities with adequate VC can be seen in Sertão Paraibano, making it the mesoregion with the largest number of municipalities with adequate VC. However, several clusters of municipalities with low VC can be observed in all mesoregions, particularly in 2017, following a line that crosses all regions.

In Fig 4A and 4B, the analysis of poliomyelitis shows clusters of municipalities with adequate VC in Sertão Paraibano, although with a visible reduction in 2017. Several clusters of municipalities with low VC can also be seen in both years, and in 2017, these clusters cross the entire state and link all mesoregions.

In terms of rotavirus VC, in 2016 (Fig 4C), clusters of adequate VC can be seen in the Sertão Paraibano, Borborema, and Agreste Paraibano mesoregions, while in the Mata Paraibana mesoregion, a cluster of municipalities with low VC is observed. On the contrary, in 2017 (Fig 4D), a reduction in the number of clusters of adequate VC and the appearance of several low VC clusters can be observed, particularly in the Sertão Paraibano mesoregion.

In terms of pneumococcal VC (Fig 4E), Sertão Paraibano is the mesoregion with most municipalities with adequate VC, including a cluster formation. Clusters of adequate VC can also be seen in the southern and southeastern parts of the Borborema mesoregion, whereas Mata Paraibana shows a cluster of low VC municipalities. In 2017 (Fig 4F), a reduction in the number of adequate VC clusters can be observed in the Sertão Paraibano and Borborema mesoregions, but new ones appeared in Agreste Paraibano and in the coastal area of Mata Paraibana.

The analysis of meningococcal VC in 2016 (Fig 4G) shows a cluster of municipalities with adequate VC in the Sertão Paraibano and Borborema mesoregions. In 2017 (Fig 4H), clusters of municipalities with low VC can be observed in all mesoregions, and there is a reduction in the number of clusters with adequate VC.

**Fig 5:**
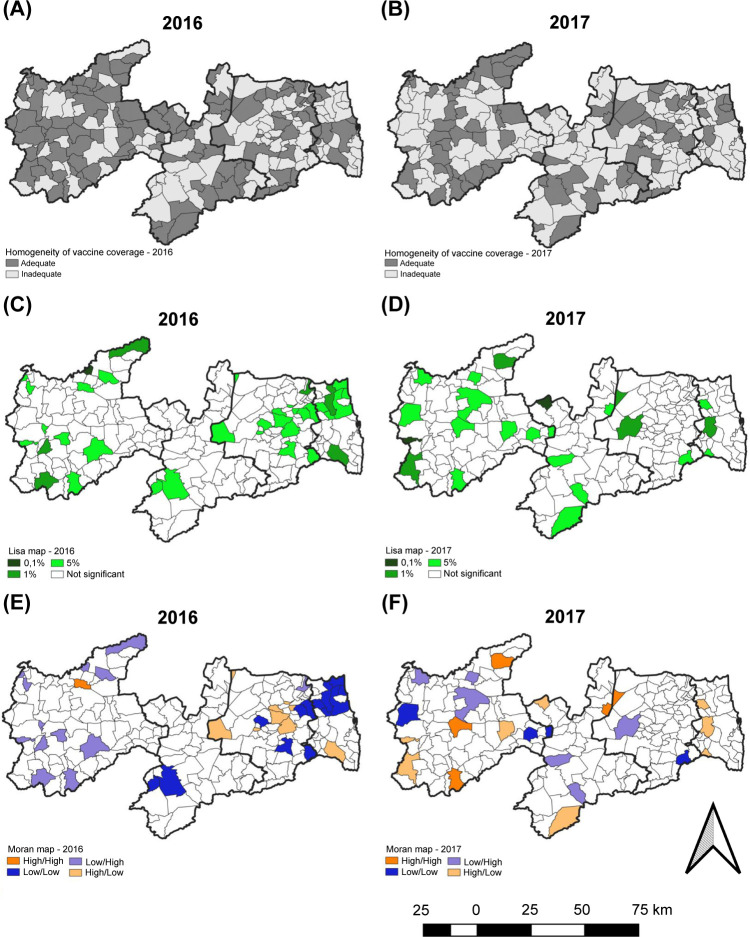
Spatial analysis of homogeneity of vaccine coverage by mesoregion in the State of Paraíba, Brazil. (A), (B): homogeneity of vaccine coverage. (C), (D): Lisa map of the homogeneity of vaccine coverage. (E), (F): Moran’s map of the homogeneity of vaccine coverage.

In the HVC analysis (Fig 5A and 5B), in 2016, there were clusters of inadequate HVC in all mesoregions. In Mata Paraibana, a large cluster of inadequate HVC comprised almost the entire mesoregion. In comparison with 2017, the clusters with inadequate HVC increased in the Agreste Paraibano, Borborema, and Sertão Paraibano mesoregions, but a reduction was observed in Mata Paraibana. Only 100 municipalities in Paraíba had adequate HVC in 2016 and only 72 municipalities in 2017.

In the analysis based on the mesoregion, Mata Paraibana, Agreste Paraibano, Borborema, and Sertão Paraibano showed 20%, 39.3%, 56.8%, and 51.8% of municipalities with adequate HVC, respectively. Mata Paraibana was the mesoregion with the worst HVC and the only one with a slight decrease in this percentage between the years studied. The other mesoregions had a significant increase in the number of municipalities with inadequate HVC, indicating that HVC deteriorated in 2017.

In the 2016 spatial analysis, only 41 municipalities out of the state’s 223 presented statistical significance (Fig 5C). When the spatial autocorrelation was analyzed (Fig 5E), it was observed that only one municipality in each of the Mata Paraibana, Agreste Paraibano, and Borborema mesoregions showed high HVC without influencing these neighborhoods (high/high). Moreover, none of the mesoregions showed a cluster, and in the entire state, only 21 municipalities showed negative spatial association (municipalities with a high HVC but surrounded by municipalities with a low HVC [high/low]).

In 2017 (Fig 5D), 27 municipalities presented statistical significance in the LISA analysis, but no municipality had a high HVC (Fig 5F).

## Discussion

Stratified VC analysis during the study period reveals a considerable set of municipalities in each mesoregion associated with low or very low VC for all immunobiological agents. Similarly, the spatial VC distribution analysis of all vaccines shows VC heterogeneity in all mesoregions, with some areas forming low VC clusters, particularly in 2017. Intermunicipality HVC was found to be below the COAP target (75%) in all mesoregions of the State of Paraíba.

PNI aims to offer all quality vaccines to all children born on Brazilian territory, thereby achieving homogeneous VC in all Brazilian municipalities [27]. Thus, only high and homogeneous VC can influence the epidemiological behavior of immunopreventable diseases [4].

The importance of controlling immunopreventable diseases and, consequently, lowering child morbidity and mortality rates is unquestionable, but since 2016, Brazilian vaccination rates have decreased for all vaccines on the National Vaccination Calendar, particularly childhood vaccines, weakening PNI and causing a resurgence of diseases that had previously been controlled or eradicated from the Brazilian reality [5]. As a result, the year 2017 has been designated as having the worst VC since 2000 [28]. Thus, meeting the childhood vaccination calendar protects children from diseases preventable by vaccines and improves community health [29].

In terms of BCG, VC falls far short of the PNI targets in all mesoregions of Paraíba because there are a significant number of municipalities with very low VC, particularly in the Mata Paraibana region. It must be emphasized that the BCG vaccine must be given, preferably within the first 12 hours of life, with the newborn still in the maternity hospital. Therefore, a loss of vaccination opportunities was found when the results were analyzed.

When the DPT/HepB/HiB immunobiological combination was examined, a high percentage of municipalities fell short of the recommended VC targets in all mesoregions, particularly in Mata Paraibana, where 50% of the municipalities had very low VC in 2016 and 70% had low VC in 2017. This data is significant because DPT is considered worldwide to be a marker of service quality owing to its scheme of three injectable doses up to the 6^th^ month of life [6].

The VC analysis of the entire set of vaccines for children under 1 year of age shows a significant difference between DPT/HiB/HepB and poliomyelitis, rotavirus, and pneumococcal, indicating a loss of vaccination opportunities in the mesoregions. This finding demonstrates the importance of identifying the factors that may be influencing the fulfillment of PNI’s recommendations.

In a study of the states of Brazil’s southeast region [30], it was observed that all states showed a decrease in VC from 2016 to 2017 for the pentavalent vaccine (DPT/HepB/HiB), correlating with the data found for Paraíba.

The study by Domingues and Teixeira [31] analyzed VC in the Brazilian child population between 2002 and 2012 and evidenced a difference of 231,000 doses between the number of total BCG doses (single dose) and the third dose of DPT/HiB as well as a difference of 500,000 doses for rotavirus and pneumococcal, indicating the number of children who could not be vaccinated. That study demonstrated a loss of vaccination opportunities, which was also found in the VC analysis conducted in Paraíba based on mesoregions.

According to Nóvoa *et al*. [32], who analyzed individual VC in Brazil, the highest coverage is for BCG in the country’s northern region, with a 109.01% population coverage. This contradicts the data for Paraíba, where BCG failed to meet the target in any of the state’s mesoregions. For rotavirus and meningococcal vaccines, that nationwide study found VC rates to be lower than the recommended target at 58.57% and 61.23%, respectively; this was consistent with the study of the mesoregions of Paraíba.

In India, Khan, Shil, and Prakash [33] observed a geographic disparity in VC between districts, both for the total vaccination scheme and for individual doses of BCG, DPT, poliomyelitis, and measles; this was consistent with the data found in Paraíba for BCG, DPT, and poliomyelitis, which show VC heterogeneity between the mesoregions.

Oliveira *et al*. [34] studied eight immunobiologicals for children under 1 year of age in the northern region of Brazil, and only BCG had VC > 90%, corroborating the data found in the present study, except for BCG, which was below the recommended target in all mesoregions of Paraíba.

Barata *et al*. [35] demonstrate uneven VC among Brazilian municipalities and the presence of low VC pockets among Brazilian municipalities. Arroyo *et al*. [36] studied areas that experienced VC reductions in Brazil and demonstrated the spatial heterogeneity of those reductions. Both studies are consistent with the results found in the present study.

In the northeastern region of Brazil, the study by Nóvoa *et al*. [32]stated that the implementation of PNI in the 1994–2019 period achieved a mean VC of 73.34%. In the temporal analysis, that study observed a tendency for an increase in VC throughout the country, except in the years 2016 and 2017; this was consistent with the data found in the present study, which showed a decrease in VC in Paraíba’s mesoregions from 2016 to 2017.

Mosser *et al*. [37] conducted a mapping of the third dose of DPT in 52 African countries between 2000 and 2016, demonstrating that in most of those countries, VC is inadequate, both at the national and intraregional levels. Their study also demonstrated that evaluating nationwide estimates alone can induce evaluation errors because divergent vaccination pockets may be masked. Thus, local analyses have the capability to channel resources to ensure that children are vaccinated. The results found in that study are consistent with those found in the present study, in which VC for DPT is low and heterogeneous within the mesoregions.

In terms of the data on HVC in Brazil between 2006 and 2011, BCG had a HVC of 49.9% in 2008, whereas in 2011, the DPT/HiB and oral poliomyelitis vaccines had a HVC close to 71%, with 72% for meningococcal and 46.9% for the decavalent pneumococcal vaccine, according to Domingues and Teixeira [31]. The results of the study are consistent with the results of the present study for the years 2016 and 2017, in which all mesoregions had results far below COAP’s target (75%).

In a study of Brazilian municipalities conducted by Braz *et al*. [7], HVC was adequate according to PQA-VS in 667 municipalities (12%) and according to COAP in 2,801 municipalities (50.3%). That same study also found 327 municipalities with a HVC of zero. When the mesoregions of Paraíba were analyzed in the present study, they failed to meet COAP’s target (75%). This is confirmed by the study by Braz *et al*. [7], which found atypical VC values (very low or very high), municipalities with a HVC of zero, and many municipalities that failed to meet the recommended target.

In a study of children under 1 year of age in the Brazilian state of Roraima between 2013 and 2017 [38], it was observed that the pneumococcal vaccine was the only vaccine that met the PNI intermunicipality target of 70% HVC. In contrast, the present study results showed that the intermunicipality HVC target was not met in any of the Paraíba mesoregions.

According to Khan, Shil, and Prakash [33], spatial analysis allows for the visualization of the VC disparity situation at the inter-municipal level, thereby aiding in the development of actions in geographic units that require closer attention.

According to Maravi *et al*. [39], the evaluation of regions by spatial analysis enables the identification of clusters of lower VC and thus the planning of more targeted health interventions. According to Yourkavitch *et al*. [40], the analysis of health indicators by geographic area allows for the identification of clusters of low VC as well as the analysis of areas of high need, thereby enabling the causes of health inequalities to be addressed.

In the present study, the comparative analysis of VC revealed the differences among the Paraíba mesoregions in terms of all immunobiologicals administered to children under the age of 1 year. The results are also consistent with those found by Arroyo *et al*. [36], Barata *et al*. [35], and Ferreira *et al*. [30].

According to Brearley *et al*. [41], understanding the peculiarities of low-performing geographic pockets and the factors that stimulate low VC is essential for health policymakers and planners who want to meet VC targets.

The situation in Paraíba in the years analyzed shows that improving VC is a challenge, both the state and national levels. Thus, identifying regions with inadequate VC is essential for PNI to succeed in public health.

Teixeira and Rocha [42] stated that the 120% limit is due to possible population invasions, i.e., migration or movement of individuals between municipalities that are not identified by the information system, as there may still be records that do not correspond to the vaccinated person’s origin. The “high” category may be related to duplicate records of doses applied, an underestimated population, or an absence of records of the individuals’ place of residence [7]. Both the municipalities in the “high” category (> 120%) and those far below the target (< 50%) have values considered atypical that should be investigated [42].

Low VC, non-homogeneity of VC, and an incomplete vaccination calendar in childhood (the age group at the greatest risk for immunopreventable diseases) are findings of the present study, indicating an urgent need to develop and plan public policies to reverse the current situation in Paraíba because controlling diseases through vaccination is indisputably essential to changing the morbidity and mortality profiles, particularly in children [5].

## Conclusions

The results of the present study show that despite the benefits of immunization, both in health (reduced child morbidity and mortality) and in the economy (vaccination is recognized as a cost-effective measure), homogeneity in vaccination has not been achieved in the mesoregions of Paraíba, making the state vulnerable to immunopreventable diseases.

A spatial heterogeneity was observed, with the presence of areas with low VC and “vaccination pockets” throughout the State of Paraíba. It was found that Sertão Paraibano was the mesoregion with the highest number of municipalities with adequate VC, and Mata Paraibana, which includes the state capital city, is the mesoregion with the lowest rates of adequate VC and the worst vaccination homogeneity.

It is concluded that there is a substantial variation in immunization among the mesoregions of Paraíba, and this requires strategic planning that takes into account each region’s peculiarities. Therefore, further studies are necessary to understand the determinants of complete immunization that can be changed because such knowledge would help immunization programs meet the recommended targets, reduce immunopreventable diseases, and change the child morbidity and mortality profiles.

## References

[1] World Health Organization. Inequality monitoring in immunization: a step-by-step manual; 2019. Available from: https://apps.who.int/iris/handle/10665/329535.

[2] World Health Organization. Global vaccine action plan: monitoring, evaluation and accountability. Available from: https://www.who.int/publications/i/item/global-vaccine-action-plan-monitoring-evaluation-accountability-secretariat-annual-report-2020. Vol. 148; 2020.

[3] Ministry of Health. Brazil. National Immunization Program: 30 years. Programa Nacional de Imunização: 30. anos. p. 208; 2003. Available from: https://bvsms.saude.gov.br/bvs/publicacoes/livro_30_anos_pni.pdf.

[4] Ministry of Health. Brazil. 40 years. [Programa Nacional de Imunização (PNI Available from: https://bvsms.saude.gov.br/bvs/publicacoes/programa_nacional_imunizacoes_pni40.pdf. Vol. 236. National Immunization Program (PNI); 2013. p. 40 years. [Programa Nacional de Imunização (PNI). Vol. 40. anos].

[5] Ministry of Health. Brazil. Health Brazil 2019: an analysis of the health situation with emphasis on immunopreventable diseases and immunization. Available from: https://portalarquivos2.saude.gov.br/images/pdf/2019/dezembro/05/Saude-Brasil-2019-imunizacao.pdf. Vol. 520; 2019 [Saúde Brasil. Uma análise da situação de saúde com enfoque nas doenças imunopreveníveis e na imunização; 2019].

[6] Ministry of Health. Brazil. National immunization program. Vaccine coverage in Brazil 2010–2014. [Coberturas vacinais no Brasil 2010–2014.]; 2015. Available from: http://portalarquivos2.saude.gov.br/images/pdf/2017/agosto/17/AACOBERTURAS-VACINAIS-NO-BRASIL—2010-2014.pdf.

[7] Braz RM, Domingues CM, Teixeira AM, Luna EJ. Classification of the transmission risk of immunopreventable diseases from vaccine coverage indicators in the municipalities of Brazil. [Classificação de risco de transmissão de doenças imunopreveníveis a partir de indicadores de coberturas vacinais nos municípios brasileiros] Epidemiol e Serv Saúde Rev do Sist Único Saúde do Bras. 2016;25:745–54. 10.5123/S1679-49742016000400008 27869968

[8] Ministry of Health. Brazil. Brasília: Ministry of Health. Vol. 1520 of 30 May 2018; 2018 [internet]. Decree. [cited Jan 1 2021]. Changes Annexes XCVIII and XCIX of Consolidation Decree No. 5/GM/MS of 28 September 2017 with the inclusion of targets and indicators of the Qualification Program for Actions in Health Surveillance—PQA VS, starting from. Available from: https://www.in.gov.br/materia//asset_publisher/Kujrw0TZC2Mb/content/id/17505595.

[9] Barbieri CL, Martins LC, Pontes Y de A, Pamplona. Immunization and vaccine coverage: past, present, and future. [Imunização e cobertura vacinal: passado, presente e futuro]. Santos: Editora Universitária Leopoldianum; 2021. 221 p.

[10] Cutts FT, Claquin P, Danovaro-Holliday MC, Rhoda DA. Monitoring vaccination coverage: defining the role of surveys. Vaccine. 2016;34(35):4103–9. doi: 10.1016/j.vaccine.2016.06.053 . 27349841 PMC4967442

[11] Sato AP. How important is vaccination hesitation in the decrease of vaccination coverage in Brazil? [Qual a importância da hesitação vacinal na queda das coberturas vacinais no Brasil?]. Rev Saúde Publ. 2018;52:1–9.

[12] Joy TM, George S, Paul N, Renjini BA, Rakesh PS, Sreedevi A. Assessment of vaccine coverage and associated factors among children in urban agglomerations of Kochi, Kerala, India. J Family Med Prim Care. 2019;8(1):91–6. doi: 10.4103/jfmpc.jfmpc_276_18 . 30911486 PMC6396615

[13] Brazilian Institute of Geography and Statistics (IBGE). Cities and states of Brazil. [Cidades e estados do Brasil] [internet]. IBGE 2021. [cited Dec 2 2021]. Available from: https://cidades.ibge.gov.br/brasil/pb/panorama. In:.

[14] Paraíba. State health secretariat. In: Vol. 2023 [internet]. p. 2020–3; 2020. State Health Plan, Paraíba, Paraíba 2020 Estadual de Saúde P, editor. State Planning and Management Board, editor. João Pessoa: Paraíba State Secretariat. Available from: https://www.conass.org.br/wp-content/uploads/2021/04/PLANOS-ESTADUAL-DE-SAUDE-PB-2020-2023.pdf.

[15] Paraíba. State health secretariat. State health plan, Paraíba 2016–2019. In: Estadual de Saúde P, editor. Available from: https://www.conass.org.br/pdf/planos-estaduais-de-saude/PB_Plano%20Estadual%20de%20Saude%202016_2019.pdf. Paraíba 2016–2019 State Planning and Management Board, editor. João Pessoa: Paraíba State Secretariat; 2016.

[16] Bezerra FJ, Bernardo TR, Ximenes LJ, Junior AS. Socioeconomic profile of Paraíba. [Perfil socioeconômico da Paraíba]. Fortaleza: Banco do Nordeste do Brasil; 2015. p. 178.

[17] Silva DMOB, Filho RS. Social vulnerability in Paraíba and its spatial disparities from the social vulnerability index. [Vulnerabilidade social na Paraíba e suas disparidades espaciais a partir do IVS]. Rev Econ Reg Urbana e do Trab. 2018;7:1.

[18] Ministry of Health. Brazil. Health surveillance secretariat. Oswaldo Cruz Foundation. Spatial approaches in public health: qualification and updating on health geoprocessing series. [Abordagens espaciais na saúde pública: Série Capacitação e Atualização em Geoprocessamento em Saúde]. 2006.

[19] Chiaravalloti-Neto F. Geoprocessing and public health. [O Geoprocessamento e saúde pública]. Arq Cienc Saúde. 2016; 23:01.

[20] Morgenstern H. Ecologic studies in epidemiology: concepts, principles, and methods. Annu Rev Public Health. 1995;16:61–81. doi: 10.1146/annurev.pu.16.050195.000425 . 7639884

[21] National Health Council (Brazil). Resolution No. 466 of 12 December 2012. Brasília; 2012. Available from: https://bvsms.saude.gov.br/bvs/saudelegis/cns/2013/res0466_12_12_2012.html.

[22] National Health Council (Brazil). Resolution No. 510 of 7 April 2016. Available from: http://conselho.saude.gov.br/resolucoes/2016/Reso510.pdf.

[23] National Health Council (Brazil). Resolution No. 580 of 22 March 2018. Available from: https://conselho.saude.gov.br/resolucoes/2018/Reso580.pdf.

[24] Brazilian Institute of Geography and Statistics (IBGE). Maps: bases and referentials: cartographic bases: digital lattices: municipal. [Mapas: bases e referenciais: bases cartográficas: malhas digitais: municipal.] Brasília: IBGE; 2016.

[25] Druck S, Carvalho MS, Câmara G, Monteiro AV. Spatial analysis of geographic data. [Análise espacial de dados geográficos]. Brasília: Embrapa; 2004.

[26] Marcos CorrêaNeves MC, Ramos FR, Camargo ECG, Câmara G, Monteiro AM et al. [Exploratory spatial analysis of São Paulo socioeconomic data. [Análise exploratória espacial de dados sócio-econômicos de São Paulo]. Campinas: Embrapa Meio Ambiente; 2000.

[27] Ministry of Health. Brazil. National immunization program—vaccination. Programa Nacional de Imunizações—Vacinação.] Available from: <http://www.saude.gov.br/noticias/693-acoes-e-programas/40594-programa-nacional-de-imunizacoes-vacinacao.

[28] Stevanim LF. Timeline: vaccination in Brazil. [Linha do tempo: vacinação no Brasil]; 2019. Available from: https://radis.ensp.fiocruz.br/index.php/home/reportagem/linha-do-tempo-vacinacao-no-brasil#access-content.

[29] Cruz A. The decrease in immunization in Brazil. [A queda da imunização no Brasil]. Available from: https://portal.fiocruz.br/sites/portal.fiocruz.br/files/documentos/revistaconsensus_a_queda_da_imunizacao.pdf.

[30] Ferreira de Souza AC, Maia FR, Rosestolato Soares Gde A, Marques LM, Marques LM, Villela Mde C. Comparative analysis of pentavalent vaccine coverage among the states of the Southeast region of Brazil. [Análise comparativa da cobertura vacinal de pentavalente entre os estados da região Sudeste do Brasil] [internet]. Vol. 13. p. 43–54; 2020. Revista Saber Digital.

[31] Domingues CMAS, Teixeira AMdS. Vaccine coverage and immunopreventable diseases in Brazil in the 1982–2012 period: advances and challenges of the National Immunization Program. [Coberturas vacinais e doenças imunopreveníveis no Brasil no período 1982–2012: avanços e desafios do Programa Nacional de Imunizações]. Epidemiol Serv Saúde. 2013;22(1):9–27. doi: 10.5123/S1679-49742013000100002

[32] Nóvoa TA, Cordovil VR, Pantoja GM, Ribeiro MES, Cunha ACS, Benjamin AIM, et al. Vaccination coverage of the National Immunization Program (PNI). [Cobertura vacinal do Programa Nacional de Imunizações (PNI)]. Braz J Heal Rev. 2020;3:7863–73.

[33] Khan J, Shil A, Prakash R. Exploring the spatial heterogeneity in different doses of vaccination coverage in India. PLOS ONE. 2018;13(11):e0207209. doi: 10.1371/journal.pone.0207209 . 30485291 PMC6261550

[34] Oliveira GS, Bitencourt EL, Ferreira P, Amaral F, Vaz GP, et al. Vaccination coverage: A comparative analysis of the states of the North region of Brazil. [Cobertura vacinal: uma análise comparativa entre os estados da região Norte do Brasil]. Rev Patol Tocantins. 2020;7:14–7.

[35] Barata RB, Ribeiro MCSA, de Moraes JC, Flannery B, Vaccine Coverage Survey 2007 Group. Socioeconomic inequalities and vaccination coverage: results of an immunisation coverage survey in 27 Brazilian capitals, 2007–2008. J Epidemiol Community Health. 2012;66(10):934–41. doi: 10.1136/jech-2011-200341 . 22268129 PMC3433223

[36] Arroyo LH, Ramos ACV, Yamamura M, Weiller TH, Crispim JA, Cartagena-Ramos D, et al. Areas with declining vaccination coverage for BCG, poliomyelitis, and MMR in Brazil (2006–2016): maps of regional heterogeneity. Cad Saude Publica. 2020;36(4):e00015619. doi: 10.1590/0102-311X00015619 . 32267382

[37] Mosser JF, Gagne-Maynard W, Rao PC, Osgood-Zimmerman A, Fullman N, Graetz N, et al. Mapping diphtheria-pertussis-tetanus vaccine coverage in Africa, 2000–2016: a spatial and temporal modelling study. Lancet. 2019;393(10183):1843–55. doi: 10.1016/S0140-6736(19)30226-0 . 30961907 PMC6497987

[38] Fonseca KR, Buenafuente SMF. Analysis of the vaccination coverage of children under age one in Roraima. 2013–2017;30:e2020195. [das A coberturas vacinais de crianças menores de um ano em Roraima, 2013–2017]. Epidemiol e Serv Saúde Rev do Sist Único Saúde do Bras. 2021. 10.1590/S1679-49742021000200010 33886806

[39] Maravi ME, Snyder LE, McEwen LD, DeYoung K, Davidson AJ. Using spatial analysis to inform community immunization strategies. Biomed Inform Insights. 2017;9:1178222617700626. doi: 10.1177/1178222617700626 . 28469433 PMC5391195

[40] Yourkavitch J, Burgert-Brucker C, Assaf S, Delgado S. Using geographical analysis to identify child health inequality in sub-Saharan Africa. PLOS ONE. 2018;13(8):e0201870. doi: 10.1371/journal.pone.0201870 . 30157198 PMC6114521

[41] Brearley L, Eggers R, Steinglass R, Vandelaer J. Applying an equity lens in the decade of vaccines. Vaccine. 2013;31;Suppl 2:B103–7. doi: 10.1016/j.vaccine.2012.11.088 . 23598470

[42] Teixeira AMS, Rocha CMV. Surveillance of vaccination coverage: A methodology for detection and intervention in risk situations. [Vigilância das coberturas de vacinação: uma metodologia para detecção e intervenção em situações de risco]. Epidemiol e Serv Saúde Rev do Sist Único Saúde do Bras. 2010;19:217–26.

